# What is the impact of preserving the endothelium on saphenous vein graft performance? Comments on the ‘NO’ touch harvesting technique

**DOI:** 10.1186/s13019-021-01397-y

**Published:** 2021-03-16

**Authors:** Ninos Samano, Andrzej Loesch, Michael R. Dashwood

**Affiliations:** 1 Department of Cardiothoracic and Vascular Surgery, Örebro University, Örebro, Sweden; 2 Centre for Rheumatology, Royal Free Hospital Campus, University College Medical School, London, UK; 3 Surgical and Interventional Sciences, Royal Free Hospital Campus, University College Medical School, Pond Street, London, NW3 2QG UK

**Keywords:** Coronary artery bypass graft, Endothelium, Nitric oxide, Patency, Saphenous vein, Tissue damage

## Abstract

Saphenous veins used for coronary artery bypass surgery are subjected to considerable vascular trauma when harvested by conventional methods. This vascular damage is responsible, at least in part, for the inferior patency of the saphenous vein when compared with the internal thoracic artery. The performance of saphenous vein grafts is improved when this conduit is harvested atraumatically using the no-touch technique. There is growing evidence that the success of the no-touch technique is due to the preservation of a number of vascular structures including the endothelium, vasa vasorum and perivascular fat. There is conflicting evidence regarding the degree of endothelial damage to the endothelium of conventional versus no-touch saphenous vein grafts. In general, it has been shown that this single layer of cells lining the lumen exhibits considerable damage associated with a combination of vascular trauma and high pressure intraluminal distension. Increased platelet aggregation and thrombus formation at the exposed subendothelial membrane is due to a local reduction of endothelium-derived factors including nitric oxide. In addition, damage to the vasa vasorum of conventionally-harvested veins will reduce transmural blood flow, a condition shown to promote neointimal hyperplasia and atheroma formation. By stripping off the perivascular fat during conventional harvesting, mechanical support of the graft is reduced and the source of adipocyte-derived factors potentially beneficial for graft patency removed. While most agree that endothelial damage to the saphenous vein affects graft patency, the contribution of other tissue-derived factors affected by vascular damage at harvesting need to be considered.

## Background

The recent study by Saito et al. [1] is based on the histological examination of samples of no-touch (NT) and conventional (CV) saphenous vein (SV) grafts (SVG) obtained from patients undergoing coronary artery bypass grafting (CABG). The study was performed on a mixed selection of SVs from seven patients, two of which were post mortem. Patients were operated both on- (*n* = 4) or off-pump (*n* = 3). In two cases NT and CV SVs were matched (i.e. from the same patient) and used for immunohistochemistry and another matched pair used for scanning electron microscopy (SEM). Separate CV and NT samples (*n* = 1) were obtained that were used for both SEM and immunohistochemistry with the autopsy material being used for immunohistochemistry alone. Quantitative assessment is presented on immunohistochemistry data from *n* = 3 and SEM data on *n* = 2 patients. Based on their study the authors suggest that factors other than the endothelium may play a pivotal role in protecting NT-harvested SVGs from atherosclerosis, thus, their results showed well-preserved endothelium in the CV SVs.

We feel that some comments are required regarding various aspects of this study. Clearly, the treatment of SV samples was varied with tissue being obtained from patients of both gender who were operated either on- or off-pump with only 2 cases where NT and CV samples were matched and in other cases NT and CV veins from separate patients were used. All patients were subjected to an echograghic examination of the SV determining the size, morphology and functionality. This is seldom performed when harvesting the vein in the CV manner. The CV was connected to the femoral line and dilated with arterial pressure for 10 min is another aspect which is not performed with CV SV harvesting. Conventional veins are manually dilated with normal saline directly after harvest reaching pressures that exceed the physiological arterial pressure by several folds. It is also not clear how the NT vs CV SVs in the post-mortem biopsies were harvested as the samples in the first five patients were obtained from the redundant SV.

**Fig. 1:**
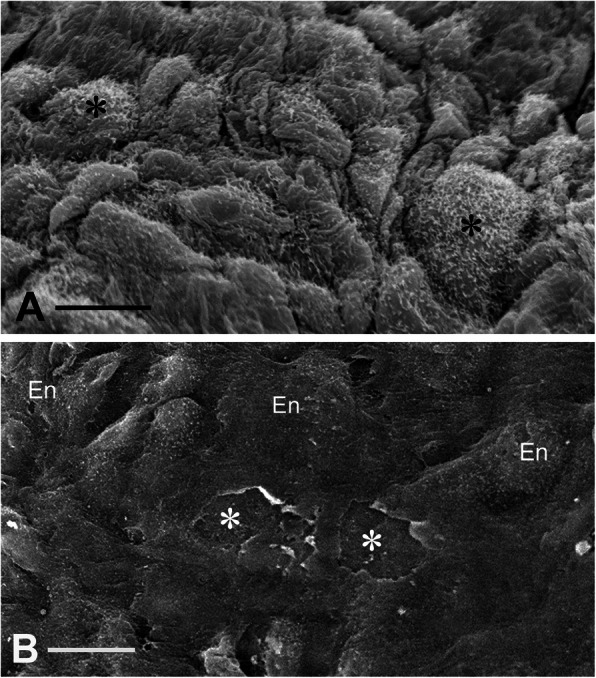
Endothelium of no-touch and conventional saphenous vein grafts for coronary artery bypass grafting. **a** No-touch saphenous vein with healthy intact endothelium (En) with the apical microvilli (asterisks). Bar: 10 μm. **b** Conventional saphenous vein with deformed En, endothelial lesions, and exposure of subendothelial connective tissue/matrix (asterisks). Bar: 20 μm. Images A and B are the adjacent segments of the SV from 59 y old male. Harvesting of B involved stripping of adiposa and partially the adventitia; distention with saline at a pressure of 300 mmHg for 1 min using a syringe connected to a manometer – a conventional harvesting lasting about 1 h 10 min. Both no-touch (**a**) and conventionally (**b**) harvested segments of SV were stored for about 30 min at room temperature in heparinized patient’s blood obtained from the aortic cannula before implantation. Samples for SEM examination were taken on completion of the proximal anastomosis and fixed and processed in the standard manner. From Vasilakis et al. 2004, which is acknowledged [4]

Regarding the results of this study, The SEM result is illustrated on ~ 64 μm^2^ of a single CV endothelial cell and 64 μm^2^ of a NT cell. For more accurate quantitative analysis, a larger intimal area, containing more endothelial cells, should be analysed. This is important as the intimal surface is certainly heterogeneous and therefore endothelial cells may display variations in microvilli due to endothelial cell age, cell regeneration etc. For reliable analysis it is also crucial that larger intimal areas of CV and NT veins are studied in order to identify regions of endothelial denudation – and that representative examples are shown. At the macroscopic level, considerable necrotic and friable tissue and diffuse atherosclerotic process was observed in CV SVG autopsy samples from CABG patients at 8 to 18 years’ post-mortem. In paired autopsy NT samples, the endothelium was well preserved with atherosclerotic process less pronounced, more localized, and organized [2]. Previous SEM studies demonstrate structural details of the intima comparing SV harvested by NT and CV techniques [3, 4]. While a healthy, intact endothelium with the apical microvilli was observed in NT SVs, the endothelium of CV SVs was deformed with endothelial lesions present and subendothelial connective tissue/matrix exposed [4] (Fig. 1). The possibility cannot be excluded that even small intimal lesions and exposure of the subendothelial matrix leads to serious pathological consequences [5]. Overall, every SVG for CABG is likely to be susceptible to injury associated with the harvesting procedure used as well as factors related with patients’ medical history. Therefore, any reduction in the mechanical trauma caused at harvesting the better will be the outcome in terms of long term graft patency [6].

The illustrations of CV and NT SV immunostaining provided by Saito et al. [1] use fluorescence with certain images barely visible, thereby reducing their impact. In addition, the CV examples seem to be presented as whole transverse sections with indications of adventitial damage. The NT examples, however, appear to be of half transverse sections that also exhibits considerable adventitial damage. Providing illustrations of stained underlying tissue would have been useful in order to show tissue histology and to assess vascular damage. In fact, the NT and CV examples provided by Saito and colleagues appear to contradict data from previously published studies since theirs show that the NT is more damaged than CV SVs. Interesting, but not discussed, is the von Willebrand Factor (vWF) staining in the adventitia, presumably of the vasa vasorum endothelium. While the authors maintain that the SV was isolated along with 5 mm of the surrounding fat tissue, this is not supported by the examples illustrated. Indeed, if the perivascular adipose tissue (PVAT) had been intact one would expect to observe vWF staining of PVAT capillary endothelium, as previously shown using CD31 to identify endothelial cells [7]. Conventional SVs were compared with NT on autopsy samples using immunohistochemistry where one patient was operated on- and the other off-pump. Clearly, post mortem tissue is, by definition, dead tissue. One questions how post mortem delay, autolysis, graft removal and storage might affect the results?

**Fig. 2:**
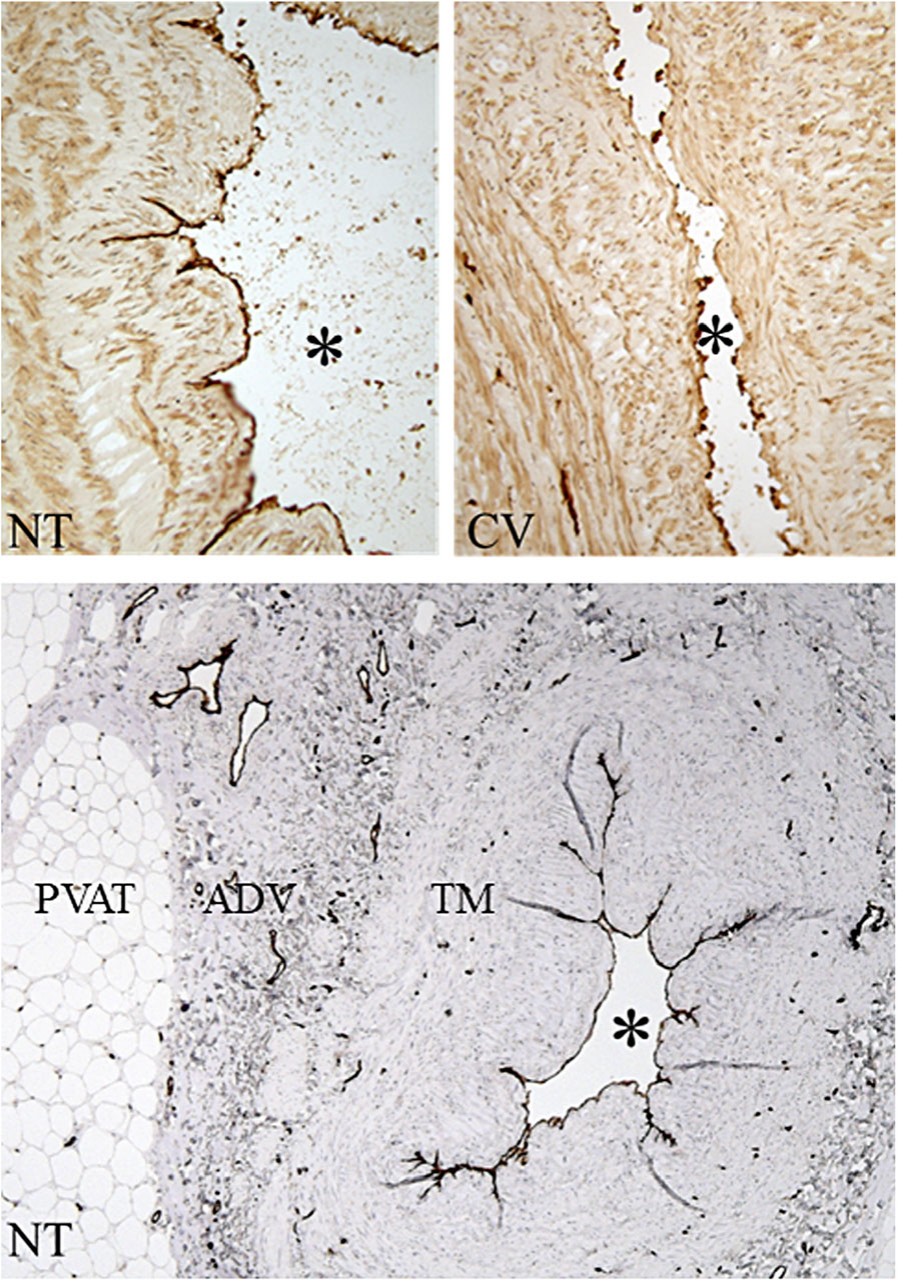
CD31 immunostaining (dark brown) of the endothelium of no-touch and conventional saphenous veins. **a** Top panels show a folded intima of a non-distended NT SV with an intact endothelium. **b** The intima of a CV SV has collapsed due to the high pressure distension used at harvesting with various regions of endothelial denudation evident. **c:** Shows a transverse section of a NT SV with intense staining of endothelial cells both of the lumen (_*_) and the vasa vasorum located within the tunica media (TM), adventitia (ADV) and capillaries within the perivascular adipose tissue (PVAT). From Dashwood et al. 2013, which is acknowledged [9]

It is surprising that no significant endothelial damage was observed in this study, particularly as distention-induced endothelial damage to CV SVs used in CABG has been described by various other groups. For example, Chester et al. [8] showed that the degree of endothelial damage was associated with the intraluminal pressure used when distending with saline. Our own work provides evidence of severe endothelial injury caused by saline distention at 300 mmHg, a ‘manual’ pressure well within the range used on CV SVs during routine CABG (Fig. 2) [10, 11]. As would be expected, endothelial cells of both the lumen and vasa vasorum express endothelial nitric oxide synthase (eNOS), the enzyme responsible for the generation of nitric oxide (NO) [12]. This has been shown on SVs obtained from CABG patients where (eNOS)/NO levels are reduced in CV vs NT SV using a combination of immunohistochemistry and tissue extracts [10, 11]. A reduction in local, endothelium-derived, NO levels will lead to an increased platelet aggregation at the exposed subendothelium, thrombus formation and early graft failure. In addition, the reduction in NO of CV SV may result in increased vascular smooth muscle cell (VSMC) proliferation, neointimal hyperplasia (NIH) and atheroma formation [13, 14]. Regarding the effects of distension often used at harvesting, based on their results, Gurkan et al. [15] showed that a pressure of 300 mmHg abolished both contraction and relaxation as a result of damage to both the endothelium and the media. Indeed, so striking were their results that the authors state, “*We consider that pressures of 300 mmHg or more turn the SVG into a dead pipe*”. In fact, a number of studies, including our own, revealed structural and immunohistochemical changes to VSMCs of the media and the vasa vasorum of the damaged adventitia of CV-harvested SV for CABG [16–19]. The impact of removing the pedicle of perivascular fat from the SV has also been discussed in terms of the possible detrimental effects on SVGs harvested conventionally [19, 20]. Regarding the authors’ statement “that factors other than the endothelium may play a pivotal role in protecting NT-harvested SVGs from atherosclerosis” – we wholeheartedly agree. While the focus of their study is the luminal endothelium they fail to consider the potential significance of the immunostaining of the endothelium of the vasa vasorum as mentioned previously. This is surprising since they cite the publication of Dreifaldt et al. [21] where the effect of vein harvesting on the vasa vasorum was studied. As a microvascular network providing the vessel wall with oxygen and nutrients, the vasa vasorum is more important in veins than in arteries. The vasa vasorum is more pronounced in veins than in arteries and penetrates deeper into the media with a suggestion that a proportion terminate in the vessel lumen [3, 18, 19, 21, 22]. While the vasa vasorum of NT remain intact, due to this atraumatic method of harvesting, the vasa vasorum in CV SVs is severely damaged, particularly at the media/adventitia border [16]. As a result, transmural blood supply will be reduced, a situation that has been shown to cause medial ischaemia, promoting neointimal hyperplasia and atherosclerotic lesion formation [23]. There is also the possibility that disruption of the vasa vasorum affects transmural transport of fat-derived factors beneficial for graft performance [19, 24].

Preservation of the cushion of fat surrounding NT SVs plays an important role in the improved performance of these grafts. For example, this PVAT possesses mechanical properties that prevents grafts of excessive length from kinking [25]. Also, this fat cushion has been shown to protect the luminal endothelium against 300 mmHg distension pressure and will therefore provide protection of the endothelium once subjected to increased coronary artery pressure at completion of CABG [11]. In addition, there is growing evidence for the role of adipocyte-derived relaxing factors [20, 24] and that such factors may be beneficial in conduits used in CABG. For example, an anti-contractile effect of PVAT has been demonstrated for both the internal thoracic artery (ITA) and SV in vitro [26, 27]. This has been supported in tissue extracts and by histology where relaxing factors such as NO, leptin and adiponectin have been identified in PVAT of NT SV used in CABG [24].

## Conclusions

We concur with the statement of Saito and colleagues (2020) “that factors other than the endothelium may play a pivotal role in protecting NT-harvested SVGs”. However, in our opinion, certain aspects of their study does not support the suggestion that “The morphological integrity of the endothelium was successfully preserved in SVG with CV”. Other features playing an important role in the improved performance of NT SV include the preservation of the medial and adventitial vasa vasorum, the mechanical properties of the intact PVAT surrounding the SV and the potential beneficial role of PVAT-derived anti-contractile factors. Many surgeons still appear unaware of the detrimental effects of the vascular damage inflicted when preparing the SV using ‘traditional’ CV harvesting. The harvesting technique of NT SVs has been shown to improve graft performance when compared with CV SVs at up to 16 years follow-up with a patency comparable to the ITA [2]. No-touch SV harvesting has now been adopted by many surgeons worldwide and was recently recognized as a class IIa recommendation in the 2018 ESC/EACTS Guidelines on myocardial revascularization [28].

## Data Availability

Not applicable.

## Abbreviations

ADV: Adventitia
CABG: Coronary artery bypass graft
CV: Conventional
En: Endothelium
eNOS: Endothelial nitric oxide synthase
ITA: Internal thoracic artery
NIH: Neointimal hyperplasia
NO: Nitric oxide
NT: No-touch
PVAT: Perivascular adipose tissue
SEM: Scanning electron microscopy
SV: Saphenous vein
SVG: Saphenous vein graft
TM: Tunica media
VSMC: Vascular smooth muscle cell
vWF: Von Willebrand factor

## References

[1] Saito T, Kurazumi H, Suzuki R, Matsuno Y, Mikamo A, Hamano K (2020). Preserving the endothelium in saphenous vein graft with both conventional and no-touch preparation.

[2] Samano N, Geijer H, Liden M, Fremes S, Bodin L, Souza D (2015). The no-touch saphenous vein for coronary artery bypass grafting maintains a patency, after 16 years, comparable to the left internal thoracic artery: a randomized trial. J Thorac Cardiovasc Surg.

[3] Souza DS, Christofferson RH, Bomfim V, Filbey D (1999). "No-touch" technique using saphenous vein harvested with its surrounding tissue for coronary artery bypass grafting maintains an intact endothelium. Scand Cardiovasc J.

[4] Vasilakis V, Dashwood MR, Souza DS, Loesch A (2004). Human saphenous vein and coronary bypass surgery: scanning electron microscopy of conventional and ‘no-touch’vein grafts. Vasc Dis Prev.

[5] Saemisch M, Balcells M, Riesinger L, Nickmann M, Bhaloo SI, Edelman ER (2019). Subendothelial matrix components influence endothelial cell apoptosis in vitro. Am J Phys Cell Phys.

[6] Samano N, Dashwood M, Souza D (2018). No-touch vein grafts and the destiny of venous revascularization in coronary artery bypass grafting—a 25 th anniversary perspective. Ann Cardiothorac Surg.

[7] Loesch A, Dashwood MR (2018). Nerve-perivascular fat communication as a potential influence on the performance of blood vessels used as coronary artery bypass grafts. J Cell Commun Signal.

[8] Chester AH, Buttery LD, Borland JA, Springall DR, Rothery S, Severs NJ (1998). Structural, biochemical and functional effects of distending pressure in the human saphenous vein: implications for bypass grafting. Coron Artery Dis.

[9] Dashwood MR, Tsui JC (2013). ‘No-touch’saphenous vein harvesting improves graft performance in patients undergoing coronary artery bypass surgery: a journey from bedside to bench. Vasc Pharmacol.

[10] Tsui JC, Souza DS, Filbey D, Karlsson MG, Dashwood MR (2002). Localization of nitric oxide synthase in saphenous vein grafts harvested with a novel "no-touch" technique: potential role of nitric oxide contribution to improved early graft patency rates. J Vasc Surg.

[11] Dashwood MR, Savage K, Tsui JC, Dooley A, Shaw SG, Fernandez Alfonso MS (2009). Retaining perivascular tissue of human saphenous vein grafts protects against surgical and distension-induced damage and preserves endothelial nitric oxide synthase and nitric oxide synthase activity. J Thorac Cardiovasc Surg.

[12] Vallance P, Moncada S (1994). Nitric oxide-from mediator to medicines. J R Coll Physicians Lond.

[13] Herbaczynska-Cedro K, Lembowicz K, Pytel B (1991). NG-monomethyl-L-arginine increases platelet deposition on damaged endothelium in vivo. A scanning electron microscopic study. Thromb Res.

[14] Colotti C, Vittorini S, Ottaviano V, Maltinti M, Angeli V, Del Ry S (2009). Nitric oxide treatment reduces neo-intimal formation and modulates osteopontin expression in an ex-vivo human model of intimal hyperplasia. Cytokine.

[15] Gurkan S, Gur O, Yuksel V, Tastekin E, Huseyin S, Gur DO (2014). The effect of distension pressure on endothelial injury and vasodilatation response in saphenous vein grafts: conversion of a bypass graft to a dead pipe. Kardiochir Torakochirurgia Pol.

[16] Ahmed SR, Johansson BL, Karlsson MG, Souza DS, Dashwood MR, Loesch A (2004). Human saphenous vein and coronary bypass surgery: ultrastructural aspects of conventional and "no-touch" vein graft preparations. Histol Histopathol.

[17] Loesch A, Dashwood MR, Souza DS (2006). Does the method of harvesting the saphenous vein for coronary artery bypass surgery affect venous smooth muscle cells? iNOS immunolabelling and ultrastructural findings. Int J Surg.

[18] Dreifaldt M, Souza D, Bodin L, Shi-Wen X, Dooley A, Muddle J (2013). The vasa vasorum and associated endothelial nitric oxide synthase is more important for saphenous vein than arterial bypass grafts. Angiology.

[19] Loesch A, Dashwood MR (2018). Vasa vasorum inside out/outside in communication: a potential role in the patency of saphenous vein coronary artery bypass grafts. J Cell Commun Signal.

[20] Fernandez-Alfonso MS, Gil-Ortega M, Aranguez I, Souza D, Dreifaldt M, Somoza B (2017). Role of PVAT in coronary atherosclerosis and vein graft patency: friend or foe?. Br J Pharmacol.

[21] Dreifaldt M, Souza DS, Loesch A, Muddle JR, Karlsson MG, Filbey D (2011). The "no-touch" harvesting technique for vein grafts in coronary artery bypass surgery preserves an intact vasa vasorum. J Thorac Cardiovasc Surg.

[22] Dashwood MR, Anand R, Loesch A, Souza DS (2004). Hypothesis: a potential role for the vasa vasorum in the maintenance of vein graft patency. Angiology.

[23] Barker SG, Tilling LC, Miller GC, Beesley JE, Fleetwood G, Stavri GT (1994). The adventitia and atherogenesis: removal initiates intimal proliferation in the rabbit which regresses on generation of a ‘neoadventitia’. Atherosclerosis.

[24] Fernandez-Alfonso MS, Souza DS, Dreifaldt M, Dashwood MR (2016). Commentary: perivascular fat and improved vein graft patency in patients undergoing coronary artery bypass surgery. Curr Vasc Pharmacol.

[25] Rueda F, Souza D, Lima Rde C, Menezes A, Johansson B, Dashwood M (2008). Novel no-touch technique of harvesting the saphenous vein for coronary artery bypass grafting. Arq Bras Cardiol.

[26] Gao YJ, Zeng ZH, Teoh K, Sharma AM, Abouzahr L, Cybulsky I (2005). Perivascular adipose tissue modulates vascular function in the human internal thoracic artery. J Thorac Cardiovasc Surg.

[27] de Vries MR, Quax PHA (2018). Inflammation in Vein Graft Disease. Front Cardiovasc Med.

[28] Neumann FJ, Sousa-Uva M, Ahlsson A, Alfonso F, Banning AP, Benedetto U (2019). 2018 ESC/EACTS Guidelines on myocardial revascularization. Eur Heart J.

